# Rsk2 inhibition induces an aneuploid post-mitotic arrest of cell cycle progression in osteosarcoma cells

**DOI:** 10.1038/s41420-025-02596-5

**Published:** 2025-07-10

**Authors:** Armelle Carreau, Christina Baldauf, Lena Warlich, Magdalena Weingartner, Laura Brylka, Michael Amling, Thorsten Schinke, Julia Luther

**Affiliations:** 1 Department of Osteology and Biomechanics, University Medical Center Hamburg-Eppendorf, Martinistraße 52, Hamburg, Germany; 2 Institute of Plant Sciences and Microbiology, University of Hamburg, Ohnhorststrasse 18, Hamburg, Germany

**Keywords:** Bone cancer, Cell death

## Abstract

Osteosarcoma is the most common primary bone tumor, which is associated with a high mortality rate. The *c-Fos* transgenic mouse model has been described to spontaneously develop osteosarcoma, and the ribosomal S6 kinase 2 (Rsk2) was found to be essential for c-Fos-induced osteosarcoma formation in mice. By isolating and characterizing osteosarcoma cell lines from *Fos*Tg and *Fos*Tg;*Rsk2*^−/y^ mice, we observed that Rsk2 deficiency impairs the growth advantage of *Fos*Tg cells. This can be explained by the aberrant number of nuclei due to impaired cytokinesis, inducing mitotic catastrophe. We therefore tested a pharmacological Rsk inhibitor (BI-D1870) for its ability to inhibit the proliferation of osteosarcoma cells and found that the effects observed by genetic Rsk2 inactivation were mimicked. BI-D1870 administration to *Fos*Tg cell lines led to reduced expression of Aurora kinase B. Therefore, the influence of a pharmacological Aurora kinase B inhibitor (Hesperadin) was tested. Similar to BI-D1870, Hesperadin caused impaired cytokinesis, resulting in the accumulation of polynuclear cells. This effect was also observed for two human osteosarcoma cell lines, U2OS and SaOS-2. Based on our findings, Rsk2 and/or Aurora kinase B can serve as potential targets for the design of new osteosarcoma therapies.

## Introduction

Osteosarcoma (OS) is the most common primary bone tumor with an incidence of 3.4 cases per million [1]. It primarily affects children and adolescents from 5 to 20 years of age. Based on metastases development or non-resectability of the tumors, the 5-year overall survival rate is still below 30% [2]. In fact, despite the development of surgery techniques and the release of new chemotherapy drugs that are the current standard in OS care, the overall survival rate has not substantially changed in the last decades. In the era of personalized medicine, it is therefore important to develop targeted therapies that will optimally permit the elimination of cancer cells or compromise their growth with a minimal effect on healthy cells. The development of these new drugs is based on the genetic characterization of tumors and the identification of proteins driving OS cell proliferation, for which specific inhibitors could be designed.

Among characterized sporadic mutations, the c-Fos oncogene was found overexpressed in the majority of human OS (≈60%). The oncogenic function of c-Fos was first established when its viral homolog *v-Fos* was identified as the gene responsible for cell transformation in the Finkel Biskis Jinkins murine sarcoma virus (FBJ-MSV) [3]. Its crucial role in the transformation of the osteoblast lineage was uncovered in vivo by the injection of *v-fos* into mice, leading to OS formation [3, 4]. Moreover, a transgenic mouse model, in which c-Fos was expressed under the control of the H-2kB class I promoter, displayed OS development throughout the skeleton, with the first tumors being detectable at 3 weeks of age [5, 6]. At a molecular level, c-Fos is one of the genes encoding a subunit of the AP-1 transcription factor, which is composed of a Fos family member (c-Fos, Fra1, Fra2, or FosB) heterodimerizing with a member of the Jun family (c-Jun, JunB, or JunD). It is noteworthy that a function in bone remodeling has been shown for many AP-1 components using genetically modified mouse models [7, 8]. However, OS development was only observed in c-Fos-transgenic mice (thereafter termed *Fos*Tg mice).

Regulation and stabilization of c-Fos are based on a series of post-translational modifications mediated by two major kinases, Erk and Rsk2, which phosphorylate c-Fos on serine 374 and 362, respectively, to prevent its degradation [9, 10]. Rsk2 is a serine/threonine kinase that belongs to the Rsk (90 kDa ribosomal S6 kinase) protein family composed of four homologs (Rsk1, Rsk2, Rsk3, and Rsk4). Its activation follows the Ras-promoted kinase cascade and leads to the regulation of multiple transcription factors participating in cell proliferation, survival, and differentiation [11]. Although reported to be overexpressed in various cancers, the role of Rsk2 in bone was primarily uncovered by the genetic examination of patients with Coffin–Lowry syndrome, an X-linked disease caused by loss-of-function variants in *RPS6KA3*, the gene encoding Rsk2. Affected patients develop severe mental retardation associated with skeletal abnormalities, growth impairment and craniofacial dystrophies which illustrates the crucial role of Rsk2 in bone development [12, 13]. A previous study conducted with *Fos*Tg mice demonstrated that Rsk2 is required for OS development in vivo. In these mice, Rsk2 deficiency reduced tumor burden without impacting OS incidence, thereby underlining the pharmaceutical potential of Rsk2 inhibition to limit OS growth [14].

The present study was conducted to understand the mechanisms involved in the limitation of tumor growth observed upon genetic inactivation of Rsk2 by analyzing OS cells isolated from *Fos*Tg;*Rsk2*^*−/y*^ mice. To support our respective findings treatment of OS cells with a pharmacological Rsk inhibitor was also performed. We thereby show that deficiency or pharmacological blockade of Rsk2 in OS cells induces an accumulation of polynuclear cells, which was associated with the downregulation of critical cell cycle regulators, such as Aurora kinase B.

## Results

### Rsk2 deficiency impairs OS expansion by limiting osteoblastogenesis at the tumor surface

**Fig. 1:**
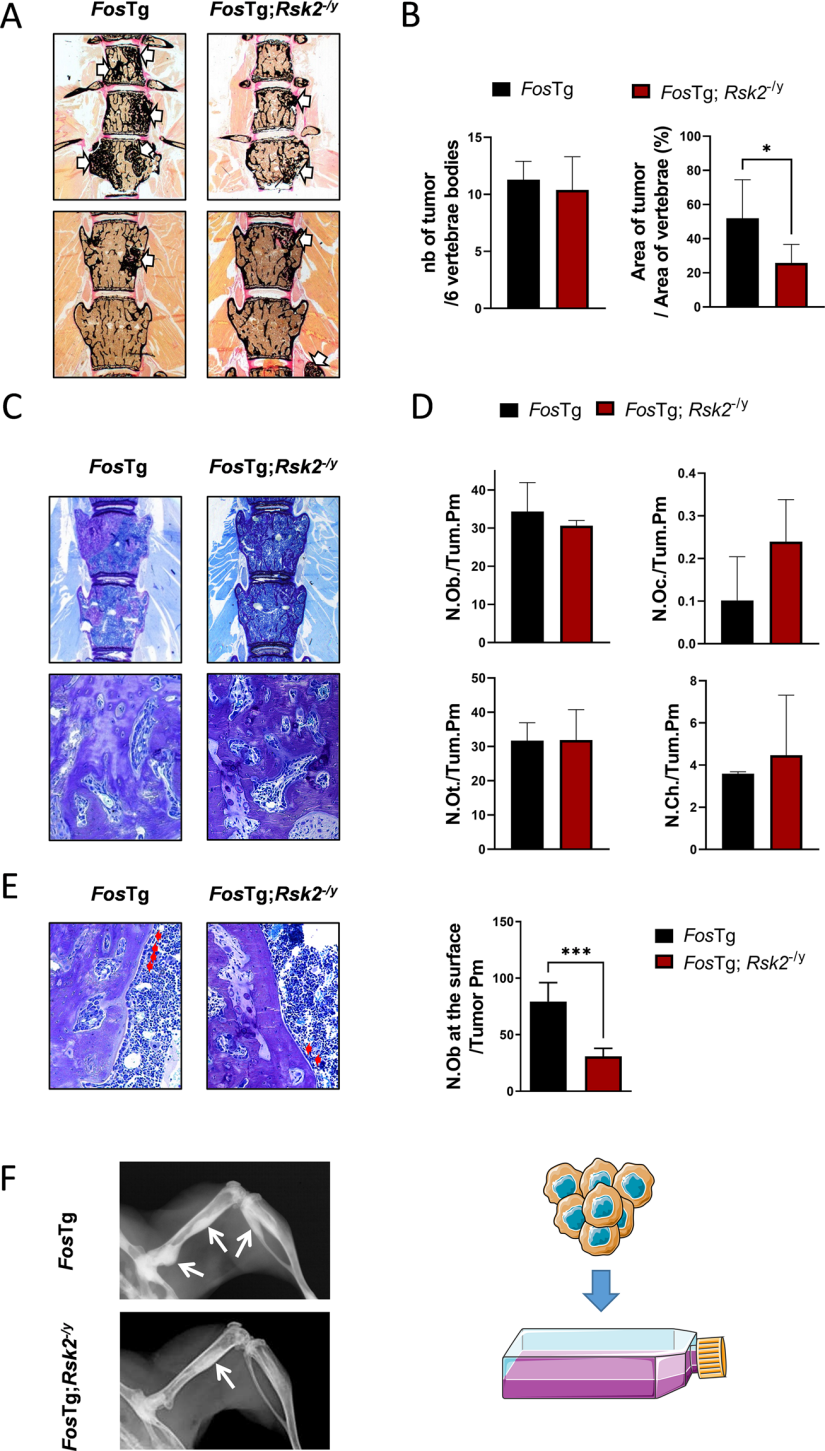
Rsk2 deficiency impairs OS expansion by limiting osteoblastogenesis at the tumor surface. **A** Representative images of undecalcified spine sections from 16-week-old mice with the indicated genotypes after Kossa-staining. Tumors are marked with white arrows. **B** Quantification of the number (nb) of tumors in spine sections (lumbar vertebral bodies L1–L6) from mice with the indicated genotypes (nb of tumor/6 vertebrae bodies) and quantification of tumor size by measuring the ratio of the area of each tumor on vertebral body area (*n* = 7–8 mice per genotype). **C** Representative images of osteosarcomas on undecalcified spine sections from mice with the indicated genotypes after staining with toluidine blue. **D** Quantification of osteoblast number (N.Ob/B.Pm), osteoclast number per bone perimeter (N.Oc/B.Pm), osteocyte number per bone perimeter (N.Ot/B.Pm) and chondrocyte number per bone perimeter (N.Ch./B.Pm) inside the tumor areas (*n* = 4 mice per genotype). **E** Representative images of osteosarcoma surfaces from mice with the indicated genotypes after staining with toluidine blue and quantification of the osteoblasts present at the tumor surface (N.Ob. at the surface/Tumor Pm), here marked with red arrows (*n* = 7–10 mice per genotype). **F** X-ray images of 16-week-old mice with the indicated genotypes. White arrows indicate the presence of osteosarcomas in the legs. Cells were extracted from these tumors and seeded in a flask to create cell lines after 10 passages. Image provided by Servier Medical Art (https://smart.servier.com/), licensed under CC BY 4.0 (https://creativecommons.org/licenses/by/4.0/). Data are expressed as mean ± SD and analyzed by unpaired two-tailed *t* test: *p < 0.05, **p < 0.01, ***p < 0.001.

It was previously reported that inactivating Rsk2 reduces OS growth in the c-Fos transgenic (*Fos*Tg) mouse model [14]. This effect was explained by decreased proliferation and increased apoptosis of tumor cells lacking Rsk2. Here we expanded this analysis by studying vertebral body sections of *Fos*Tg and *Fos*Tg;*Rsk2*^−/y^ mice, since this allowed a quantitative analysis of eight vertebral bodies per mouse (Fig. 1A). We thereby observed that the number of visible OS was not affected by the Rsk2 deficiency, but that the size of the tumors was strongly reduced (Fig. 1B). Toluidine blue staining of the sections revealed a high heterogeneity inside the OS areas, yet cellular histomorphometry did not identify specific differences between *Fos*Tg and *Fos*Tg;*Rsk2*^−/y^ littermates (Fig. 1C, D). In contrast, however, the number of osteoblasts at the tumor surfaces was drastically reduced in *Fos*Tg;*Rsk2*^−/y^ compared to *Fos*Tg mice (Fig. 1E). With the aim to understand the cellular mechanisms driving this phenotype correction, we generated OS cell lines from isolated long bone tumors of *Fos*Tg and *Fos*Tg;*Rsk2*^−/y^ mice and analyzed their behavior ex vivo (Fig. 1F).

### Rsk2 inactivation abolishes the growth advantage of *Fos*Tg OS cells

**Fig. 2:**
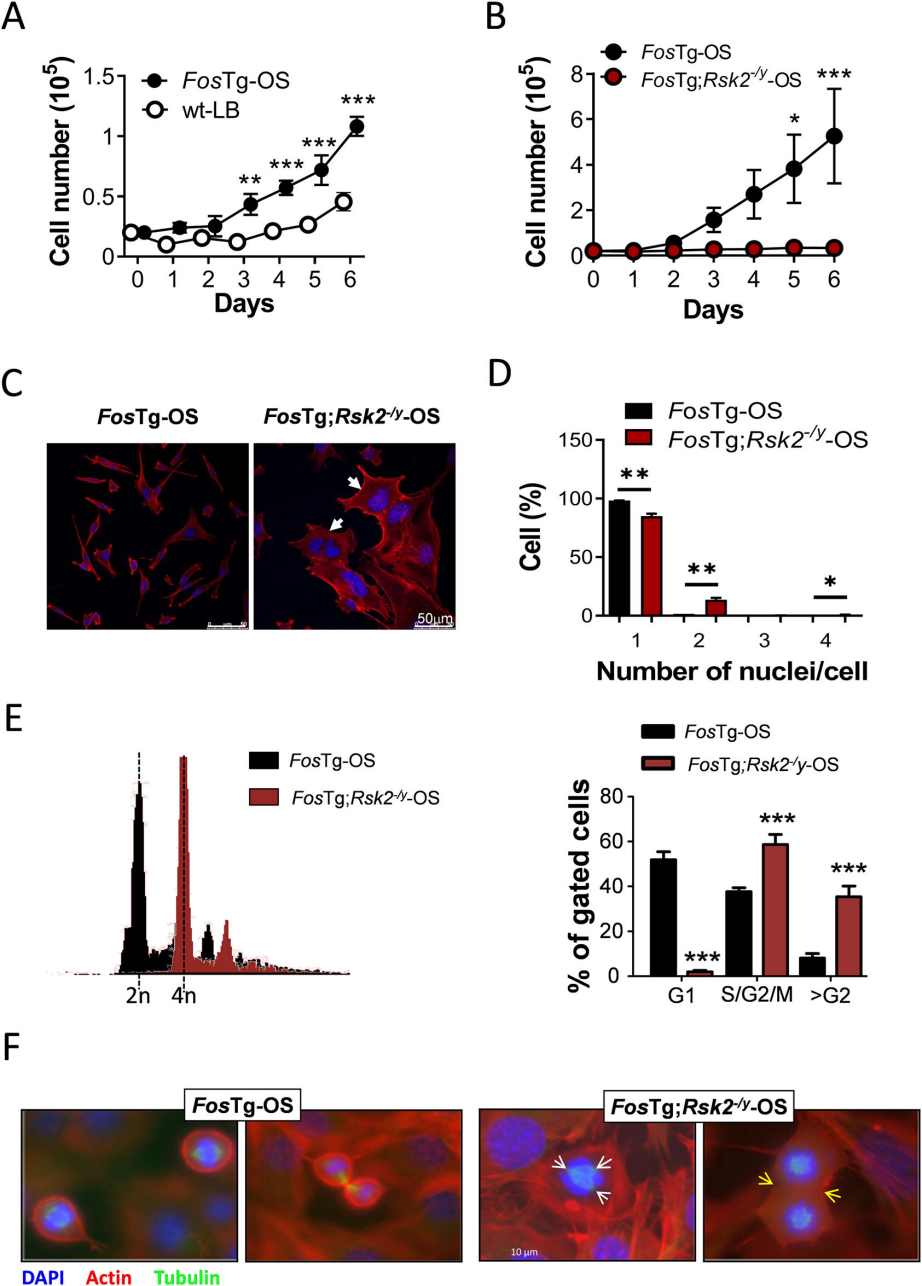
Rsk2 inactivation abolishes the growth advantage of FosTg OS cells. **A** Comparative growth curve of cells isolated from osteosarcomas growing in Fos-transgenic male mice (*Fos*Tg-OS) compared with cells isolated from long bones of age- and sex-matched wild-type mice (wt-LB) (*n* = 4 independent isolations per genotype). **B** Comparative growth curves of established cell lines (passage>12) isolated from osteosarcomas developing in the long bones of *Fos* transgenic mice and *Fos* transgenic mice lacking Rsk2 (*Fos*Tg;*Rsk2*^*−/y*^ (OS)) (*n* = 6 independent cell lines per genotype). **C** Representative images of *Fos*Tg-OS or *Fos*Tg;*Rsk2*^*−/y*^-OS cells stained with phalloidin-red for β-actin and DAPI for DNA, the white arrows indicate the presence of bi-nucleated cells. **D** Quantification of the proportion of cells with the indicated number of nuclei after 24 h of BI-D1870 treatment compared to DMSO-treated cells (*n* = 3 independent cell lines per genotype). **E** Representative FACS profiles of DNA content in cell lines of the indicated genotypes and quantification of the proportion of cells in G1, S/G2/M of the cell cycle, as well as polyploid cells (>G2) (*n* = 3 independent cell lines). **F** DAPI, Phalloidin-red and Tubulin staining of representative *Fos*Tg and *Fos*Tg;*Rsk2*^*−/y*^ cell lines. Notice the presence of multiple mitotic spindles (white arrows) and the impairment of cytokinesis (yellow arrows). The results are presented as the mean ± SD and analyzed by one-way ANOVA if one variable or two-way ANOVA if more, with *p < 0.05, **p < 0.01, ***p < 0.001.

When compared to wild-type long bone cells (wt-LB), cells isolated from OS developing in *Fos*Tg mice long bones (*Fos*Tg-OS) had a clear growth advantage at early passages, when cells are considered as primary cell culture (Fig. 2A). We therefore performed experiments comparing cells isolated from the tumors developing in Rsk2-deficient *Fos*Tg mice (*Fos*Tg; *Rsk2*^*−/y*^-OS) to cells from the tumors of *Fos*Tg mice (*Fos*Tg-OS). Here we found that genetic inactivation of Rsk2 abolishes the Fos-dependent growth advantage of the OS cells (Fig. 2B). The morphological comparison of these cells by immunofluorescence staining uncovered the appearance of polynuclear cells (up to 4 nuclei per cell) in Rsk2-deficient OS cells (Fig. 2C, D). This mitotic defect was further confirmed by flow cytometry analysis of the cell cycle profile, indicating an accumulation in M and sub-G2 phases of the *Fos*Tg;*Rsk2*^−/y^ cells compared to *Fos*Tg cells (Fig. 2E). To monitor the mitotic progression of these cells, a triple staining (DAPI for nuclei, Phalloidin red for cytoskeleton and γ-tubulin for mitotic spindle) was performed. Here, disorganized mitotic spindles and cytokinesis defects were observed in Rsk2-deficient OS cells (Fig. 2F). Together, these data suggest that the genetic inactivation of *Rsk2* abolishes the growth advantage of OS cells isolated from *Fos*Tg tumors by inducing mitotic defects leading to polyploidy.

### Pharmacological blockade of Rsk2 mimics the influences of Rsk2 deficiency on OS growth

**Fig. 3:**
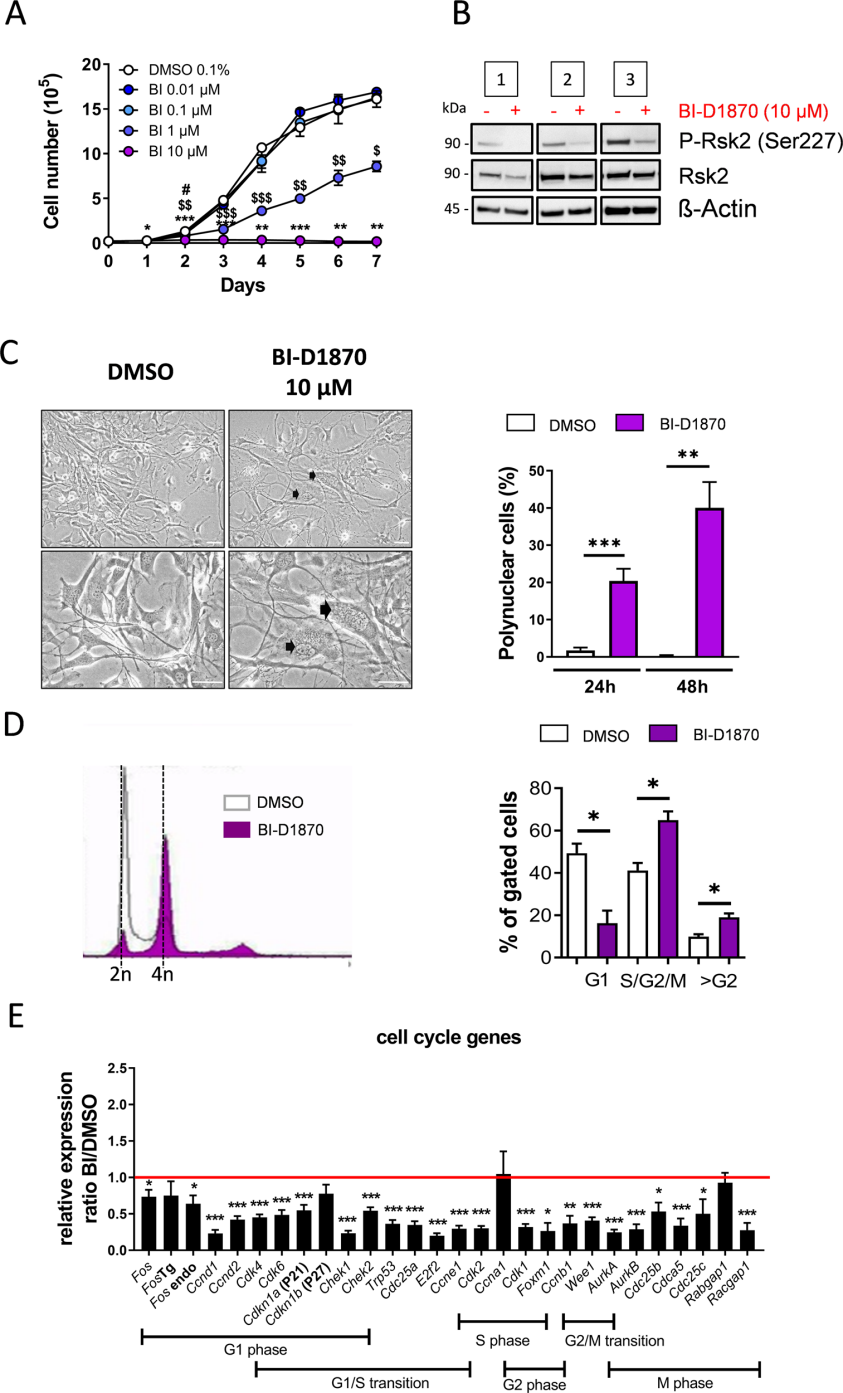
Pharmacological blockade of Rsk2 mimics the influences of Rsk2 deficiency on OS growth. **A** BI-D1870 dose-dependent (0.01–10 μM) inhibition of the growth of three independent cell lines isolated from osteosarcoma of *Fos* transgenic mice, DMSO is used as a carrier, the arrow indicates the timing of addition of the inhibitor (*n* = 3 independent cell lines). The results are presented as the mean ± SD and analyzed by Two-way ANOVA with *p < 0.05, **p < 0.01, ***p < 0.001. **B** Western blot analysis of Rsk2 and phosphorylated Rsk2 on serine 227 of three independent *Fos*Tg cell lines treated for 24 h with BI-D1870 (10 μΜ) (+) or with the carrier DMSO (−). ß-actin was used as a loading control. **C** Representative pictures of *Fos*Tg cells treated with BI-D1870 (10 μM) or with the carrier DMSO (scale bar = 25 μm). Quantification of polynuclear cells (marked here with black arrows) is shown on the right (*n* = 5 independent cell lines) with data presented as the mean ± SD and analyzed by two-tailed unpaired Student’s *t*-test. **D** Representative FACS profiles of DNA content in cell lines 24 h after addition of BI-D1870 compared to DMSO-treated cells and quantification of the proportion of cells in G1, S/G2/M of the cell cycle, as well as polyploid cells (>G2) (*n* = 3 independent cell lines). **E** Q-PCR analysis of the expression of markers of the different phases of the cell cycle 24 h after stimulating *Fos*Tg-OS cell lines with 10 μM of the Rsk inhibitor BI-D1870. The result represents the ratio of Q-PCR values of the BI-D1870 treated cells divided by Q-PCR values of the DMSO treated cells (n>5independent cell lines/gene). The results are presented as the mean ± SD and analyzed by one-sample *t*-test compared to the incubation DMSO with *p < 0.05, **p < 0.01, ***p < 0.001.

The growth and mitotic failure of Rsk2-deficient OS cells also suggested a therapeutic potential of Rsk inhibitors for OS treatment. To validate this hypothesis *FosTg* cells were treated with increasing doses of BI-D1870, a pharmacological inhibitor of Rsk isoforms Sapkota et al., [15]. Here, a dose of 10 μM was found to be the most effective, in accordance with data from other publications, to limit cellular growth by reducing Rsk2 activation (Fig. 3A, B and Original data in Supplementary Fig. S1). Morphologically, the treatment of *Fos*Tg cells with BI-D1870 resulted in a striking increase of polynuclear cells after 24 h which further rose after 48 h (Fig. 3C). The FACS analysis of the different phases of the cell cycle highlighted a blockade in the G2/M phase when cells were treated with BI-D1870, similar to the observations made by genetic inactivation of Rsk2 (Fig. 3D). To gain insight into the potential mechanism driving the growth arrest induced by the inhibition of Rsk2 we performed a qPCR analysis. Upon BI-D1870 administration, there was a significant downregulation in *Fos*Tg cells of key cell cycle markers (*Cyclin D1*, *Cdk4*, *Cdk6*, *Cdk2*) and of genes regulating cell cycle progression (*Cdkn1a*, *Cdkn1b*, *E2f2*, *Trp53*) (Fig. 3E). Notably, within this set of genes, *AurkB*, encoding for Aurora kinase B, emerged to be a salient candidate, potentially serving as a mediator of Rsk2. In fact, Aurora kinase B is known for its pivotal function within the G2/M phase transition of mitosis and in the control of cytokinesis. Furthermore, numerous inhibitors targeting Aurora kinase B are currently under clinical investigation and have been reported to induce similar mitotic defects in other cell types as observed after BI-D1870 treatment of *Fos*Tg cells [16, 17].

### Pharmacological blockade of Aurora kinase B induces polyploidy in OS cells

**Fig. 4:**
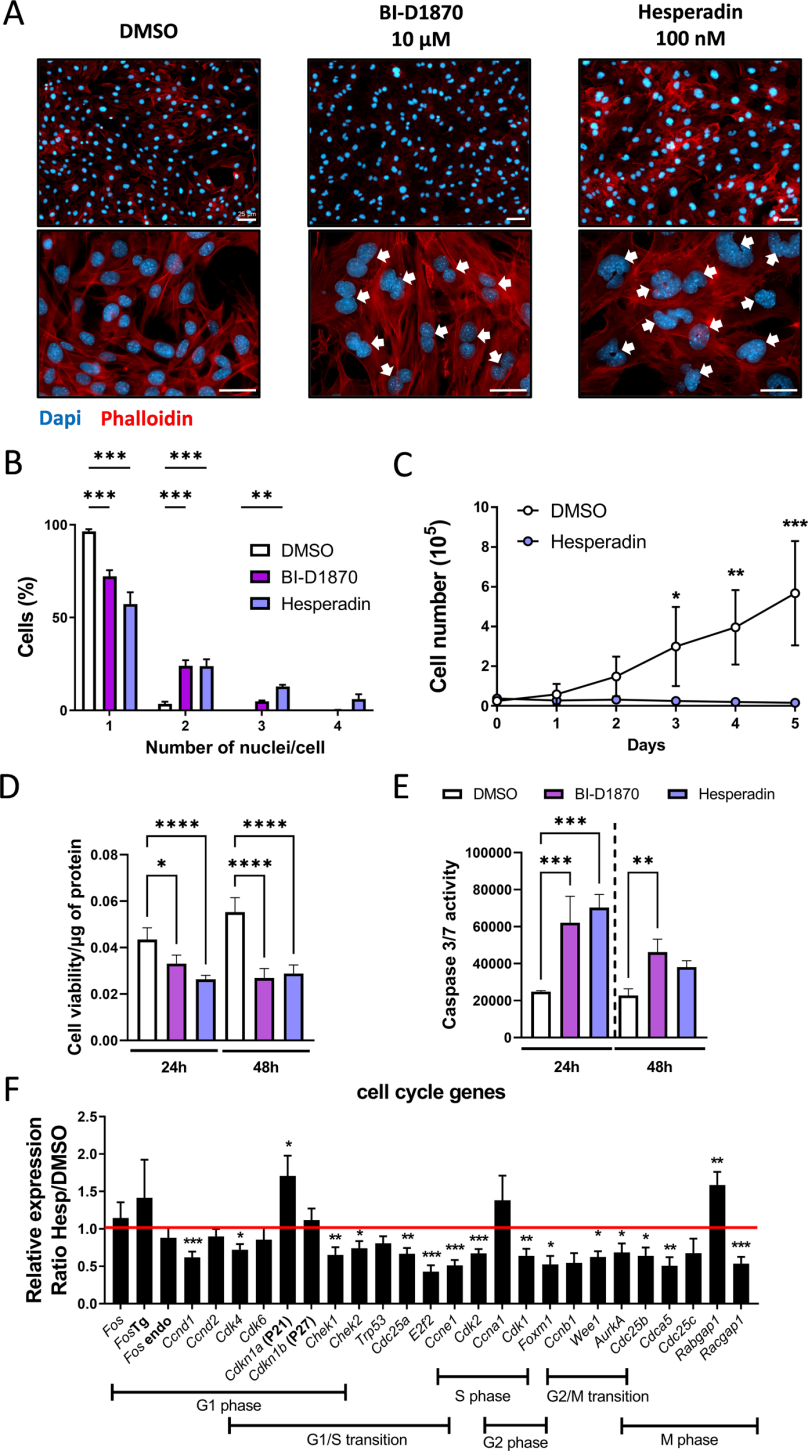
Pharmacological blockade of Aurora kinase B induces polyploidy in OS cells. **A** Representative pictures of *Fos*Tg-OS cells stained with phalloidin-red for β-actin and DAPI for DNA after treatment with 10 μM BI-D1870, 100 nM Hesperadin or DMSO (carrier), the white arrows indicate the presence of polynuclear cells (scale bar = 25 μm). **B** Quantification of the proportion of cells with more than 1 nucleus after 24 h of BI-D1870, Hesperadin treatment compared to DMSO-treated cells (*n* = 4 independent cell lines) with data presented as mean ± SD with**p < 0.01, ***p < 0.001 analyzed by two-way ANOVA. **C** Growth curves of *Fos*Tg cell lines treated with Aurora kinase B inhibitor Hesperadin (100 nM) added after 24 h of seeding compared to DMSO. Cell quantification was performed upon six consecutive days (*n* = 3 independent cell lines. **D** Cell viability, determined by MTT assay, after 24 or 48 h of treatment with BI-D1870 or Hesperadin (*n* = 3 independent cell lines). Data are presented as mean ± SD, and values were normalized to the control of each cell line (DMSO) with*p < 0.05, **p < 0.01, analyzed by One-way ANOVA. **E** Apoptosis induction analysis by luminescent quantification of caspase 3/7 activity after 24 or 48 h of cell treatment with BI-D1870 or Hesperadin. Data represent the mean ± SD ratio of caspase 3/7 activity following treatment with the different inhibitors normalized to DMSO (carrier) (*n* = 4 independent cell lines). Data were analyzed by One-way ANOVA with*p < 0.05, **p < 0.01. **F** Q-PCR analysis of the expression of markers of the different phases of the cell cycle 24 h after stimulating *Fos*Tg-OS cell lines with 100 nM of the Aurora kinase B inhibitor, Hesperadin. The result represents the ratio of Q-PCR values of the Hesperadin-treated cells divided by Q-PCR values of the DMSO-treated cells (*n* > 6 independent cell lines/gene). Values are presented as mean ± SD and analyzed by one-sample *t*-test for incubation with DMSO with *p < 0.05,**p < 0.01, ***p < 0.001.

*Fos*Tg cells were therefore treated with Hesperadin, which was first described as a potent inhibitor of Aurora kinase B in HeLa cells [18] and more recently proved its efficacy to repress pancreatic cancer growth in mice [19]. Similar to BI-D1870, Hesperadin (100 nM) treatment for 24 h led to a drastic increase in cells carrying more than 2 nuclei compared to the control (DMSO) (Fig. 4A, B). These results revealed that both inhibitors induced a defect in mitosis, leading to an impairment of cytokinesis. A further study of the growth of *Fos*Tg cells under Hesperadin treatment revealed a similar proliferation impairment as with BI-D1870 treatment (Fig. 4C). To further characterize the division impairment of *Fos*Tg cells treated with both inhibitors a MTT assay was performed to analyze cell viability. Here, we observed a significant reduction in cell viability after 24 or 48 h of treatment with both inhibitors (Fig. 4D and Supplementary Fig. S2). This decrease in cell viability was associated with the induction of apoptosis, as demonstrated by a caspase 3/7 luminescent assay. More specifically, we found that 24 h of treatment of *Fos*Tg cells with Hesperadin or BI-D1870 caused a significant increase in caspase 3/7 activity, while after 48 h, only a moderate decrease in Caspase 3/7 activity was observed following BI-D1870 treatment (Fig. 4E and Supplementary Fig. S2). Assessing the major cell cycle gene expression, particularly those associated with the G2/M phase, led to similar results as observed with BI-D1870 treatment (Fig. 4F). Taken together, we observed a comparable effect of Aurora kinase B inhibitor (Hesperadin) on *Fos*Tg cells as with BI-D1870 treatment, thus suggesting a common pathway.

### Pharmacological blockade of Rsk2 and Aurora kinase B inhibits the growth of human OS cell lines independently of the P53 status

**Fig. 5:**
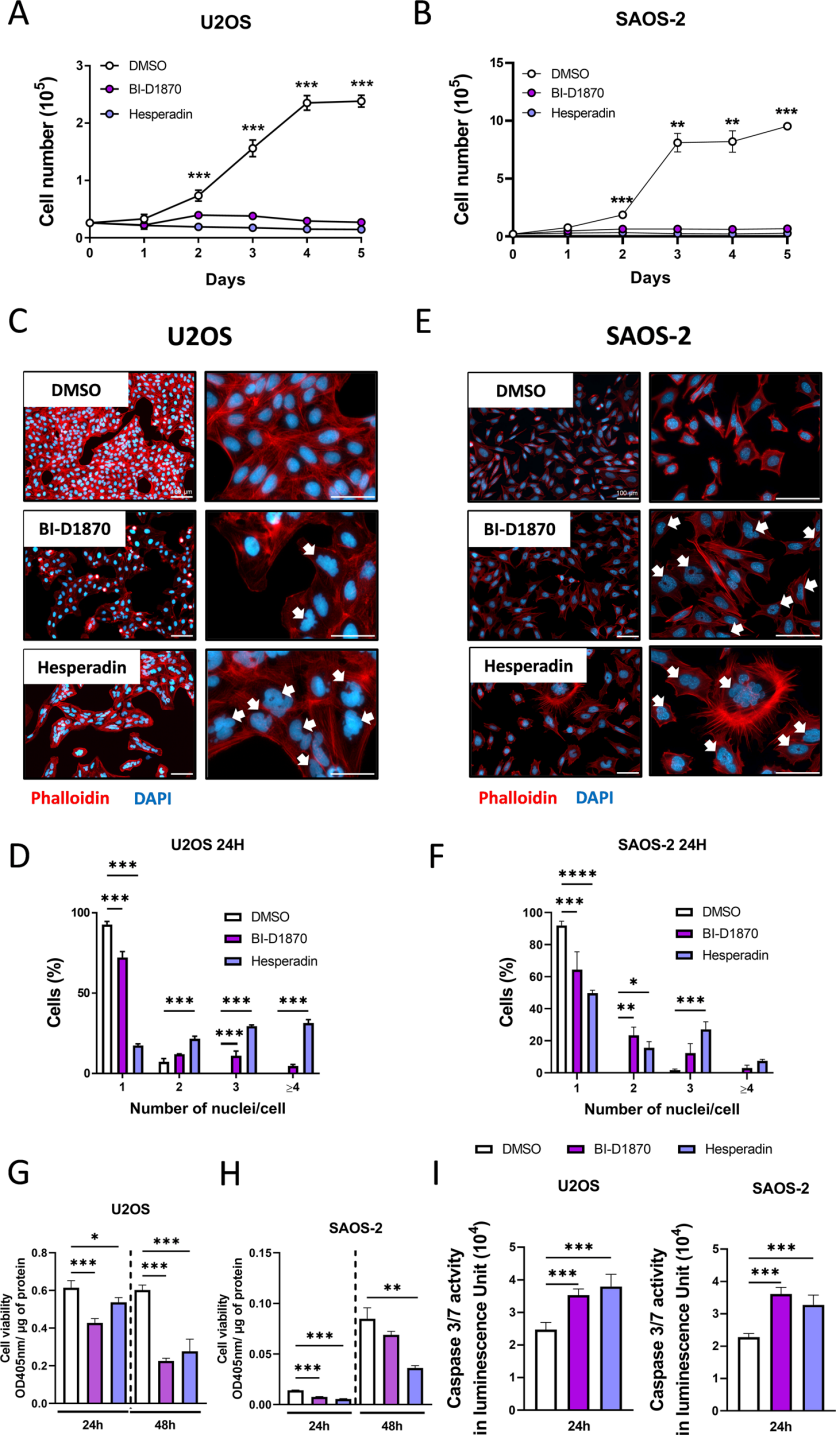
Pharmacological blockade of Rsk2 and Aurora kinase B inhibits the growth of human OS cell lines independently of the P53 status. **A** Growth curve of U2OS cells treated with 10 μM of BI-D1870 or 100 nM of Hesperadin compared to DMSO-treated cells. **B** Growth curve of the P53-deficient human OS cell line SAOS-2 treated with 10 μM BI-D1870 or 100 nM Hesperadin compared to DMSO-treated cells analyzed by Two-way ANOVA with **p < 0.01, ***p < 0.001. **C** Representative pictures of Phalloidin red and DAPI staining of the U2OS cells (a human osteosarcoma cell line) 24 h after BI-D1870 or Hesperadin treatment. White arrows indicate the presence of cells with more than one nucleus (scale bar = 100 μm). **D** Quantification of the proportion of U2OS cells with 1,2,3 or 4 nuclei (*n* = 3) after 24 h of respective treatments, done using ImageJ cell counter. **E** Representative pictures of Phalloidin red and DAPI staining of the SAOS-2 cells (a human osteosarcoma cell line) 24 h after BI-D1870 or Hesperadin treatment, the white arrows indicate the presence of cells with more than one nucleus. **F** Quantification of the proportion of SAOS-2 cells with 1,2,3 or 4 nuclei (*n* = 3) after 24 h of respective treatments, done using ImageJ cell counter. The results are presented as the mean ± SD and analyzed by Two-way ANOVA with *p < 0.05,**p < 0.01, ***p < 0.001. **G** and **H** Cell viability of U2OS and SaOS-2 cells treated for 24 h with BI-D1870 (10 μM) or Hesperadin (100 nM) was determined by MTT assay, and absorbance values were normalized to the control (DMSO) (*n* = 3 for each cell line). Values are presented as mean ± SD and analyzed by one-sample *t*-test after the incubation with DMSO with **p < 0.01, ***p < 0.001. **I** Caspase 3/7 activity of U2OS and SAOS-2 cells treated for 24 h with BI-D1870, Hesperadin or DMSO (*n* = 3 for each cell line). The results are presented as the mean ± SD and analyzed by One-way ANOVA with *p < 0.05,**p < 0.01, ***p < 0.001.

To address the question of whether the two inhibitors are also able to induce growth arrest in human OS cells, we chose two established cell lines (U2OS and SAOS-2), which are known to have different p53 status. We found that treatment of the p53-positive cell line U2OS with BI-D1870 and Hesperadin significantly limited cell growth over a period of 6 consecutive days (Fig. 5A). Similarly, growth of the reported p53-deficient cell line SAOS-2 [20] was also impaired following the treatment by both compounds, indicating that their efficacy is independent of p53 (Fig. 5B). The staining of nuclei with DAPI and cytoskeleton fibers with phalloidin revealed that both treatments lead to polyploidy in U2OS (Fig. 5C, D) and SAOS-2 (Fig. 5E, F) cells. We also found that U2OS cell viability significantly decreased in response to BI-D1870 and Hesperadin treatment after 48 h (Fig. 5G). Similar results were obtained in SAOS-2 cells, but here a more drastic reduction of cell viability was already detectable after 24 h of treatment with both BI-D1870 and Hesperadin (Fig. 5H). We also analyzed the apoptotic response and found an induction of caspase 3/7 expression in U2OS and SAOS-2 cells after treatment with BI-D1870 and Hesperadin (Fig. 5I). These findings show that both inhibitors induce a growth arrest and apoptosis in human osteosarcoma cells independent of the P53 status.

## Discussion

Our study demonstrates that the growth defect observed in FosTg-OS cells lacking Rsk2 is caused by cell-autonomous genomic instabilities associated with aneuploidy, ultimately leading to mitotic catastrophe. This effect, observed following genetic inactivation of Rsk2, was also replicated using the Rsk inhibitor BI-D1870, which downregulates *AurkB* expression. Inhibition of Aurora kinase B similarly disrupted mitosis, limiting *Fos*Tg cell growth and inducing apoptosis. These findings were validated in two established human OS cell lines, confirming the therapeutic potential of targeting this pathway.

Rsk2 is a kinase that regulates c-Fos protein levels through phosphorylation, stabilizing c-Fos and enhancing its transforming activity [9, 10, 21]. Importantly, *Rsk2* genetic inactivation has been previously described to reduce OS size in *Fos*Tg mice, which was also confirmed here [14]. Our study establishes a specific role for Rsk2 in promoting OS cell proliferation. Analysis of *Fos*Tg mice revealed that Rsk2 deficiency impairs tumor growth by reducing active osteoblasts at the tumor surface. Isolated OS cells lacking Rsk2 exhibited even more pronounced growth defects ex vivo, supporting a cell-autonomous mechanism. Previous studies have shown that Rsk2 depletion disrupts spindle assembly checkpoint signaling by impairing the kinetochore localization of key mitotic regulators such as Mad1, Mad2, and CENP-E [22]. Furthermore, Rsk2 depletion has been linked to prolonged mitotic duration and weakened kinetochore-microtubule interactions, leading to defects in chromosome alignment and mitotic spindle stability [23]. Our findings further support the role of Rsk2 as a key regulator of mitotic progression. We found that Rsk2 inactivation, whether genetic or pharmacological, disrupts OS cell growth by inducing mitotic failure and mitotic catastrophe, a process triggered by repeated mitotic defects not resolved through normal cell death pathways [24–26]. Since polyploidy is a hallmark of cancer and often associated with poor prognosis, our findings support the potential of the induction of mitotic catastrophe as a therapeutic strategy for OS [15].

To explore the therapeutic potential of Rsk2 inhibition, we treated *Fos*Tg cells with BI-D1870. Although BI-D1870 inhibits Rsk2, it also targets other Rsk isoforms (Rsk1, Rsk3, and Rsk4), which have varying roles in cancer. While some Rsk isoforms are associated with increased cancer aggressiveness [27], others, like Rsk3 and Rsk4, exhibit anti-tumoral effects in ovarian cancer [28]. Therefore, although Rsk inhibitors targeting all isoforms are in clinical trials, their role remains controversial [29, 30]. Additionally, Rsk2 inactivation has been linked to increased migration and aggressiveness in hepatocellular carcinoma [31], emphasizing the need for further investigation into its role in OS.

We demonstrated that BI-D1870 (10 μM) significantly impairs the growth of various *Fos*Tg-derived tumor cell lines by causing G2/M cell cycle arrest and mitotic catastrophe. Gene expression analysis revealed a general downregulation of cell cycle genes. BI-D1870 has previously been shown to induce apoptosis in leukemia and melanoma cells [29, 32, 33], and our study confirms its apoptotic effect in both murine and human OS cells (U2OS and SAOS-2). Similar findings have been reported in a neuroblastoma in vivo study, where BI-D1870 reduced tumor burden in mice without affecting vital organs [34]. Our results also show that *Fos*Tg cells treated with BI-D1870 lead to a downregulation of *AurkB*, which encodes a crucial enzyme for proper mitotic progression.

Aurora kinase B inhibition is known to cause aneuploidy by preventing proper cytokinesis, aligning with our BI-D1870 treatment observations. Like the Rsk family, the Aurora kinase family consists of serine/threonine kinases commonly overexpressed in cancers [35–38], and overexpression of Aurora kinase B correlates with poor prognosis in several cancers, including ovarian, osteosarcoma, thyroid, prostate cancer, and leukemia [39–44]. More specifically, Aurora kinase B is known to play a crucial role in chromosome alignment, segregation during mitosis, and cytokinesis [45]. Aurora kinase B inhibitors, such as Barasertib, have shown promise in clinical trials, reducing tumor growth and delaying relapse in patients with advanced cancers [46, 47].

To further validate this pathway, we treated *Fos*Tg cells with the Aurora kinase B inhibitor Hesperadin, which produced similar effects as BI-D1870. Our results also show that Rsk2 and Aurora kinase B regulate apoptosis, as their inhibition led to caspase 3/7 activation in *Fos*Tg cells. While a direct link between Rsk2 and Aurora kinase B in caspase regulation remains unclear, previous studies indicate that Aurora kinase B inhibition increases caspase 3 cleavage [48, 49]. Additionally, Rsk2 has been shown to phosphorylate and inactivate BAD, preventing cytochrome C release and caspase 3/7 activation, suggesting that its inhibition could enhance apoptosis in OS cells [50]. This suggests that both kinases may cooperate to suppress apoptosis. These findings support the idea that dual inhibition of Rsk2 and Aurora kinase B, or their combination with chemotherapy, may represent an effective therapeutic strategy for OS patients. While our study assessed apoptosis through caspase 3/7 activity, two key executors of this process, previous research has reported that Aurora kinase B knockdown can also trigger autophagy, a distinct caspase-involved form of programmed cell death, resulting in reduced migratory and invasive potential of osteosarcoma cells [51]. This mechanism warrants further investigation in our model.

To assess the therapeutic potential of these inhibitors in OS patients, we treated two human OS cell lines (U2OS and SAOS-2) with BI-D1870 and Hesperadin. Despite their different p53 status (wildtype in U2OS, deficient in SAOS-2 [20]), both cell lines responded similarly, suggesting that these inhibitors act independently of p53. Since P53 mutations are present in 80–90% of OS cases and are associated with poor prognosis [52], these results are particularly important. They highlight that both inhibitors can effectively inhibit OS cell proliferation, regardless of P53 expression. In fact, while a previous study has shown that Aurora kinase B inhibitors can induce apoptosis in U2OS cells [44], our study emphasizes that this effect is P53-independent.

In conclusion, our study supports the potential of Rsk2 and Aurora kinase B inhibitors, such as BI-D1870 and Hesperadin, as promising therapeutic options for OS. These inhibitors induce mitotic catastrophe and apoptosis in OS cells independent of P53 status. Many cancer patients receive targeted therapies against upstream regulators of Rsk2, such as EGFR, BRAF, or MEK inhibitors [53–56]. While these treatments improve survival, drug resistance remains a major challenge, often requiring combination therapies. Additionally, their broad impact on cellular pathways can lead to significant side effects, highlighting the need for more specific downstream targets. Single or dual inhibition of RSk2 or Aurora kinase B could offer a novel therapeutic approach for OS, particularly in P53-deficient cases. These findings warrant further investigation in clinical trials to assess their potential in overcoming resistance and improving treatment outcomes.

## Materials and methods

### Mice and genotyping

The *Fos*Tg mouse model was generated by placing the proto-oncogene c-*Fos* under the control of an H-2K^b^ class I MHC promoter [5]. *Rsk2*^−/y^ mice have been described in [14]. Mice were maintained on a C57BL/6J background. Genotyping was performed by PCR using the primers listed in Table 1.

**Table 1:** List of genotyping primers.

Mouse model	Forward primer	Reverse primer	Band size (bp)
*Fos*Tg	AGTCTGGCCTGCGGGTCTC	GTCGGCTGGGGAATGGTAGTAGG	600
Rsk2^−/y^	TTGTTGGTTTACTTTCTTTCGGTCTG	AAGATGATTGCTTTGCTTAGTTTA	230 (WT) 320 (KO)

### Ethics approval

All animal experiments were approved by the animal facility of the University Medical Center Hamburg-Eppendorf and by the “Amt für Gesundheit und Verbraucherschutz” (application numbers: Org984, N19/053_Metabone, N59/22). Mice's health status was monitored daily, and mice were analyzed at an age where the animals were not severely affected and if showing early signs of pain, loss of weight, motricity or behavior changes, sacrificed instantly.

### Histological analysis and histomorphometry

For undecalcified histology, lumbar vertebral body samples were dehydrated in increasing concentration of ethanol (80–100%) and embedded in methylmethacrylate following a previously described protocol [57]. Sections of 4 μm thickness were cut in the sagittal plane with a Microtec rotation microtome (Techno-Med GmbH, DE). Von Kossa/van Gieson and Toluidine blue (Sigma Aldrich Corp., St. Louis, US) stainings of lumbar vertebral sections were performed according to standard protocols. The tumor area was then defined using ImageJ software by performing a ratio of the tumor surface to its vertebral body surface. Measurements were made on four vertebral bodies per mouse, and the median of tumor area/vertebral body area was collected on a graph. Cellular content quantification was performed by histomorphometry analysis on Toluidine blue-stained vertebral sections using an OsteoMeasure system (Osteometrics Inc., GA, USA). Bone cell quantification of tumors was based on the measurement of the number of osteoblasts per bone perimeter (N.Ob/B.Pm), number of osteocytes per bone perimeter (N.Ot/B.Pm), osteoclasts per bone perimeter (N.Oc/B.Pm), number of chondrocytes per bone perimeter inside the tumors (N.Ch./B.Pm), osteoblasts at the surface of the tumor per tumor bone perimeter (N.Ob/B.Pm) according to ASBMR guidelines [58].

### Cell isolation: Bone marrow, long bone and tumor

Tumoral and long-bone cells were isolated from dissected tumors or flushed long bones from mice as follows: the tumors or long bones were minced with scissors, the pieces recovered in α-MEM medium complemented with 10% fetal bovine serum (FBS), collected by centrifugation and plated in six-well plates. The media was changed every 2–3 days until a layer of cells had grown out of the bone pieces. Daily growth curves were obtained by plating cells at early passage (*p* < 4) or late passage (*p* > 12) in 12-well plates at 20,000 cells per well.

### Cell culture and treatment

Human osteosarcoma cell lines, U2OS (#HTB-96) and SAOS-2 (#HTB-85), were purchased from ATCC (Manassas, VA, USA). They were cultured as instructed in McCoy 5a supplemented with 1% Penicillin/Streptomycin (Thermo Fisher Scientific Inc., Waltham, USA) and 10% or 20% FBS (Gibco, Life Technologies Corp., New York, USA), respectively. *Fos*Tg cells were maintained in α-MEM medium supplemented with 10% FBS and 1% penicillin/streptomycin. Cells were incubated at 37 °C with 5% CO_2._ For Rsks inhibition experiments, cells were treated with BI-D1870 (Calbiochem®, Sigma Aldrich) for 24 and 48 h in their standard medium. For Aurora kinase B inhibition experiments, cells were treated with Hesperadin (Selleck Chemicals GmbH, Houston, USA) for 24 and 48 h in their standard medium.

### Immunofluorescence microscopy

For in vitro immunofluorescence staining, cells were seeded at a density of 5000 cells/well on chamber slides (Ibidi GmbH) and treated with either BI-D1870 (10 μM), Hesperadin (100 nM) or DMSO (carrier). After removing the medium, cells were washed with PBS and fixed with 4% PFA for 10 min at room temperature. Cells were then washed three times with ice-cold PBS. Phalloidin Alexa fluor 546 nm (Invitrogen) was added diluted 1:40 in antibody dilution buffer (10 ml PBS, 30 μl Tween 20, 10 mg BSA) and incubated at room temperature for 90 min protected from light. After washing with PBS, nuclei were stained with DAPI (2.5 μg/ml) diluted 1:250 in PBS. Pictures were taken with a Zeiss ApoTome (UKE Microscopy Imaging Facility). Tubulin and actin staining were performed on coverslips, cells were fixed in 4% PFA and incubated with 0,1% Triton before blocking in FBS. Phalloidin A546 nm was used to mark actin and a Tubulin primary antibody (#2125, Cell Signaling Technology, MA, USA), prelabeled using a Zenon^TM^ mouse IgG1 kit (Invitrogen, Massachusetts, USA) was used for Tubulin detection. For visualizing Rsk2 localization during mitosis of *Fos*Tg cells a Rsk2-antibody conjugated to Alexa fluor 647 (sc-9986 AF647 from Santacruz 1:100) was used.

### Cell cycle progression analysis by fluorescence-activated cell sorting (FACS)

For FACS analysis, osteosarcoma cell lines treated with BI-D1870 or DMSO were used. The staining was performed on 1.5 × 10^6^ cells that were fixed with 70% EtOH for 15 min on ice. After 5 min centrifugation at 1500 rpm and 4 °C, the resulting cell pellet was resuspended in 500 μl propidium iodide (PI) solution (Sigma Aldrich Corp.), a fluorescent dye that intercalates into double-stranded DNA and incubated for 40 min at 37 °C in the dark. The RNase contained in the staining solution prevented the binding of PI to RNA. After a washing step, the stained cells were resuspended in 500 μl PBS and measured in a FACS Calibur flow cytometer (Becton, Dickinson and Co., New Jersey, USA). Data acquisition was carried out using CellQuestTM software. The DNA content and cell cycle phase distribution could be analyzed using the histograms created.

### RNA extraction, RT-qPCR

RNA of BI-D1870 or Hesperadin-treated cells was isolated using Trifast reagent (Peqlab, US). For qRT-PCR expression analysis, RNA was reverse transcribed using the Verso cDNA synthesis kit (Thermo Fisher Scientific, Massachusetts, USA) according to the manufacturer's instructions. A stepOneplus system and predesigned Taqman gene expression assays (Thermo Fisher Scientific) or designed SYBR green with self-designed primers have been used for a quantitative expression analysis (Applied Biosystems, Massachusetts, USA). Data analysis was performed according to the delta-delta comparative threshold cycle (2ΔΔCt) method, and relative gene expressions were normalized against a housekeeping gene: glyceraldehyde 3-phosphate dehydrogenase (*Gapdh*). Results are shown as fold-change expression values relative to control (treated with Dimethyl sulfoxide, Carl Roth GmbH & Co.KG, Karlsruhe, DE). The lists of primers used are included in Tables 2 and 3.

**Table 2:** List of Taqman® assay probes.

Genes	Taqman® probe
*AurkA*	Mm01248177_m1
*AurkB*	Mm01718146_g1
*Ccnb1*	Mm03053893_gH
*Ccnd1*	Mm00432358_g1
*Ccnd2*	Mm00438070_m1
*Ccne1*	Mm00432367_m1
*Cdc25b*	Mm00499136_m1
*Cdca5*	Mm01233533_m1
*Cdk1*	Mm00772472_m1
*Cdk2*	Mm00443947_m1
*Cdk4*	Mm00726334_s1
*Cdk6*	Mm01311342_m1
*Cdkn1a* (P21)	Mm04205640_g1
*Cdkn1b* (P27)	Mm00438168_m1
*Chek1*	Mm001176757_m1
*Chek2*	Mm00443844_m1
*E2f2*	Mm00624964_m1
*Foxm1*	Mm00514924_m1
*Gapdh*	4308313
*Rabgap1*	Mm01284516_m1
*Racgap1*	Mm00488847_m1
*Trp53*	Mm01731287_m1
*Wee1*	Mm00494175_m1

**Table 3:** List of sybrgreen primers.

Genes	Forward primer sequence	Reverse primer sequence
*Ccna1*	GGAAATTGCAGCTTGTCGGG	GGTGGTTGGAACGGTCAGAT
*Cdc25a*	CTCAGAAGCTCCTGGGATGTAG	GAGATGCAGGTCGTATTGGCT
*Cdc25c*	TACCATCCGTTCAGATTTCCC	TCCTCAAGGTCAGCAGAAGT
*cFos* endo	TGTGTTCCTGGCAATAGCGTGT	TGAACATTGACGCTGAAGGAC
*cFos*Tg	TGTGTTCCTGGCAATAGCGTGT	GGCAATTCCGCCCATAGTGA
*Gapdh*	GACATCAAGAAGGTGGTGAAGCAG	CTCCTGTTATTATGGGGGTCTGG

### MTT assay

For the cell viability assay, 10,000 cells/wells were seeded in opaque-walled 96-well plates (Thermo Fisher Scientific). *Fos*Tg cells were treated with DMSO, BI-D1870 (10 μM) or Hesperadin (100 nM) in standard growth medium containing 10% FBS and 1% Penicillin/Streptomycin. After 24 and 48 h of treatment, cell viability was measured by performing an MTT assay, incubating the cells for 3 h in their growth medium without FBS but supplemented with 1 mg/ml of Thiazolyl blue-Tetrazolium bromide powder (Sigma Aldrich Corp.). After solubilization in Isopropanol of the crystals, the absorbance at 570 nm was measured (background 650 nm).

### Apoptosis assay

The level of apoptotic cells was quantified by measuring *Fos*Tg OS or wildtype cells' caspase 3–7 activities by bioluminescence using the Caspase Glo-3/7 assay according to manufacturer instructions (G8090; Promega, Madison, WI, USA). Measurements were performed following 24 or 48 h of treatment with BI-D1870 (10 μM), Hesperadin (100 nM) or DMSO.

### Western blot analysis

Cells were seeded at a density of 400,000 cells per tissue culture dish (100 × 20 mm from Sarstedt, DE). After 24 or 48 h of treatment, protein extraction was performed on non-confluent cells with RIPA lysis buffer (10 mM Tris–HCl, 1 mM EDTA, 0.5 mM EGTA, 1% Triton X-100, 0.1% Sodium Deoxycholate, 0.1% SDS, 140 mM NaCl, pH = 8) supplemented with phosphatase and protease inhibitors (Roche Applied Science, Penzberg, DE) for 15 min at 4 °C, followed by a centrifugation step (15 min, 4 °C, 14,000 rpm). After quantification of protein concentration by Bradford assay (Bio-rad Laboratories, USA), 20 μg of protein were separated on mini gel (Bolt™ 12%, Bis–Tris, 1.0 mm from Life Technologies, California, USA) for SDS–PAGE analysis. The transfer was performed on nitrocellulose membranes. Membranes were blocked for 1 h in TBS-T supplemented with 5% BSA and incubated overnight with antibodies anti-β-actin (#5732 1:1000), Rsk2 (#5528 1:1000) or phosphorylated Rsk2 on serine 227 (#3556 1:1000 in TBS-T 5% BSA), all obtained from Cell Signaling Technology (Massachusetts, USA). HRP-conjugated secondary antibody (#P0448 Dako/Agilent Technologies Inc., USA) was used. Revelation after adding Pierce™ ECL Western Blotting Substrate solution was performed on hyperfilms (GE Healthcare Bio-Sciences AB, SE).

### Statistical analysis

We performed an unpaired two-tailed Student’s *t* test to compare the significance between two groups, and One-way ANOVA or Two-way ANOVA Bonferroni’s multiple comparisons test to compare significance among more than two groups with one or several variables. *P* value of ≤0.05 was considered significant, and expressed as ns > 0.05, *P ≤ 0.05, **P ≤ 0.01, ***P ≤ 0.001, ****P ≤ 0.0001. The error bars represent mean ± SD. The data analysis was performed using Prism 9 statistical software (GraphPad Software Inc., San Diego, CA, USA).

## Supplementary information

Supplementary Figure S1

Supplementary Figure S2: Proliferation and apoptosis analysis of FosTg cells treated with BI-1870 and Hesperadin.

Supplementary Figure S2 legend: Proliferation and apoptosis analysis of FosTg cells treated with BI-1870 and Hesperadin.

## Data Availability

Research data are stored in an institutional repository and will be shared upon reasonable request to the corresponding author.
