# Supplementary material for: Rsk2 inhibition induces an aneuploid post-mitotic arrest of cell cycle progression in osteosarcoma cells
Source: Cell Death Discov. 2025 Jul 10;11:318. doi: 10.1038/s41420-025-02596-5 (PMC12241552; doi:10.1038/s41420-025-02596-5)

Supplementary Figure S1: Original Western Blot data

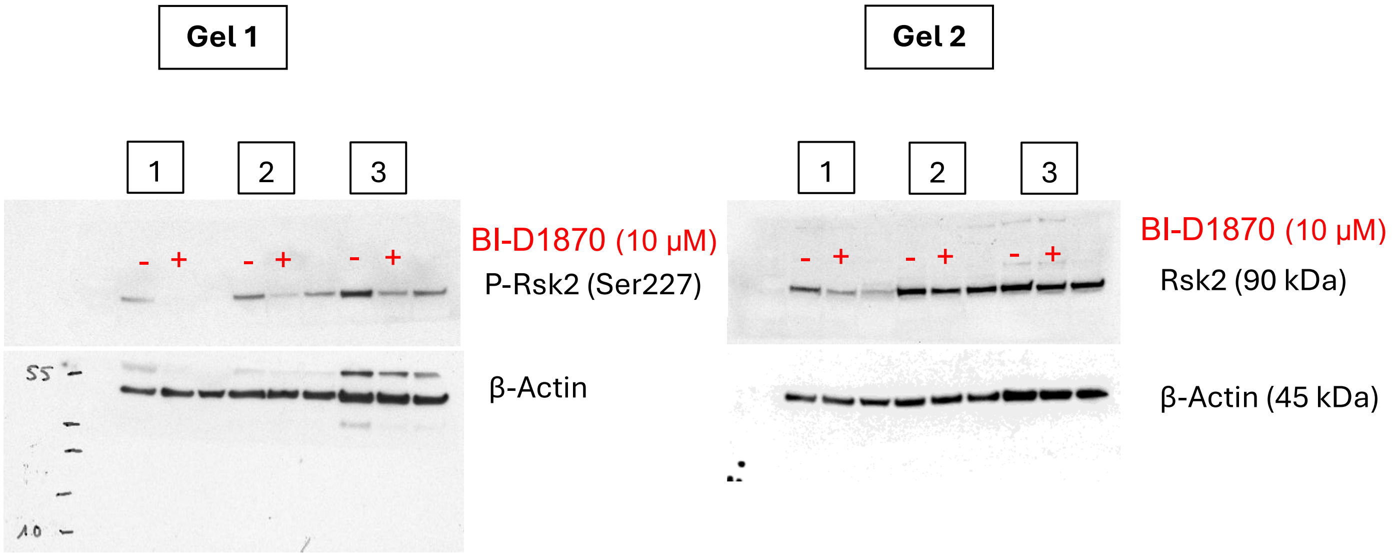

Supplement: Supplementary file 1 — Supplementary Figure S1 [file 41420_2025_2596_MOESM1_ESM.pdf]
