# Supplementary figures and images for: Rsk2 inhibition induces an aneuploid post-mitotic arrest of cell cycle progression in osteosarcoma cells
Source: Cell Death Discov. 2025 Jul 10;11:318. doi: 10.1038/s41420-025-02596-5 (PMC12241552; doi:10.1038/s41420-025-02596-5)

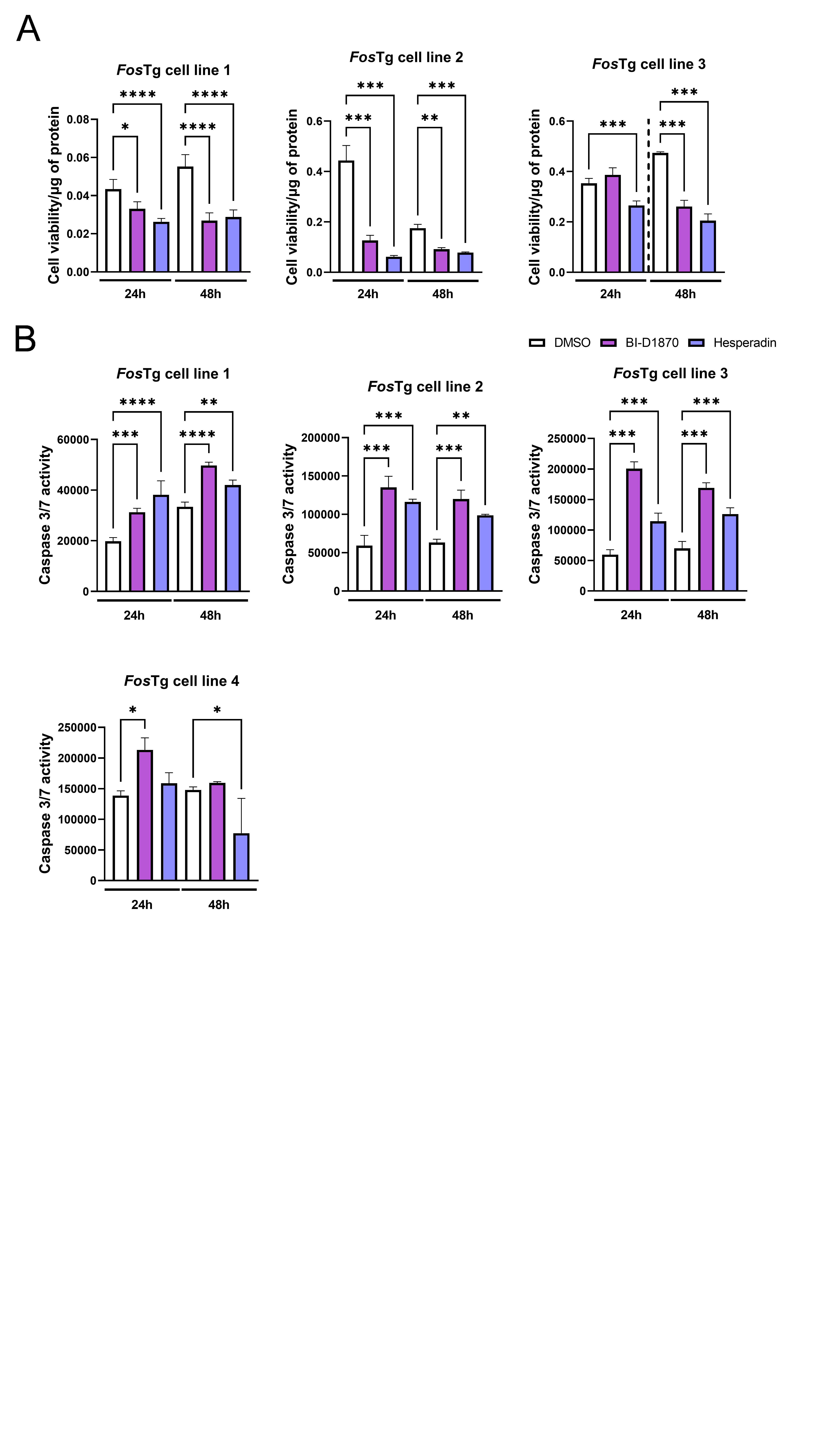

Supplement: Supplementary file 2 — Supplementary Figure S2: Proliferation and apoptosis analysis of FosTg cells treated with BI-1870 and Hesperadin. [file 41420_2025_2596_MOESM2_ESM.png]
