# Supplementary material for: Rsk2 inhibition induces an aneuploid post-mitotic arrest of cell cycle progression in osteosarcoma cells
Source: Cell Death Discov. 2025 Jul 10;11:318. doi: 10.1038/s41420-025-02596-5 (PMC12241552; doi:10.1038/s41420-025-02596-5)
Supplement: Supplementary file 3 — Supplementary Figure S2 legend: Proliferation and apoptosis analysis of FosTg cells treated with BI-1870 and Hesperadin. [file 41420_2025_2596_MOESM3_ESM.pdf]

Supplementary Figure S2: Proliferation and apoptosis analysis of *Fos*Tg cells treated with BI-1870 and Hesperadin.

A) Cell viability of each cell line (n=4 independent *Fos*Tg cell lines) presented in individual graphs measured in quadruplicates and determined by MTT assay, after 24 or 48 hours of treatment with BI-D1870 or Hesperadin with datas presented as mean ±SD with*p<0.05, **p<0.01, ***p<0.001 analyzed by One-way ANOVA. **B)** Apoptosis induction analysis by luminescent quantification of caspase 3/7 activity after 24 or 48 hours of cell treatment with BI-D1870 or Hesperadin. Data are presented as individual graphs for each cell line (n=4 independent *Fos*Tg cell lines) which have been measured in triplicates and represent the mean ±SD of caspase 3/7 activity following treatment with the different inhibitors or DMSO (carrier). Data were analyzed by One-way ANOVA with *p<0.05, **p<0.01, ***p<0.001.
